# DO PSYCHOLOGICAL AND SOCIAL FACTORS MODERATE LINKS BETWEEN CARDIOVASCULAR HEALTH AND COGNITION IN LATER LIFE?

**DOI:** 10.1093/geroni/igz038.2404

**Published:** 2019-11-08

**Authors:** Jeremy B Yorgason, Melanie S Hill, Hailey Wellar, Lance Erickson, Shawn Gale

**Affiliations:** Brigham Young University, Provo, Utah, United States

## Abstract

Cardiovascular health is related to cognition in later life (Samieri, 2018). Psychological factors, such as depressive symptoms, have been linked with cardiovascular health (Thomas, Kalaria, & O’Brien, 2004). Marital quality, an important indicator of social connection, has been linked with cardiovascular response (Seider et al., 2009), and both depression and marital satisfaction are linked with a quicker recovery from heart attacks (Keller, 1998). Depressive symptoms and marital quality may buffer links between cardiovascular health and cognitive functioning. The purpose of this study was to examine cardiovascular links with cognition, in connection with depressive symptoms and marital quality. Using data from 864 participants of the Life and Family Legacy study (Mean age = 61.78), we examined predictors from 2010 in relation to cognition measured in 2017/2018. Word recall and computation subscales of the Minnesota Cognitive Acuity Screen (MCAS) were used to assess cognitive functioning. Results from multiple regression models indicated that after controlling for age, gender, education, income, and marital status, having hypertension and higher depressive symptoms were predictive of word recall. Lower depressive symptoms were also predictive of higher computation scores. Depression did not moderate links between cardiovascular health and cognitive functioning. Among married participants (n=632), positive marital quality had no main effect nor moderating association with cardiovascular health predicting cognitive functioning. Further research is needed to better understand how biological, psychological, and social factors interact to affect cognition in later life. Longitudinal work should track these associations in context of cognitive changes with aging.

